# Can Music Influence Patients With Disorders of Consciousness? An Event-Related Potential Study

**DOI:** 10.3389/fnins.2021.596636

**Published:** 2021-04-09

**Authors:** Yajuan Hu, Fengqiong Yu, Changqing Wang, Xiaoxiang Yan, Kai Wang

**Affiliations:** ^1^Department of Neurology, The First Affiliated Hospital of Anhui Medical University, Hefei, China; ^2^Department of Medical Psychology, Chaohu Clinical Medical College, Anhui Medical University, Hefei, China; ^3^Collaborative Innovation Center of Neuropsychiatric Disorders and Mental Health, Hefei, China; ^4^Anhui Province Key Laboratory of Cognition and Neuropsychiatric Disorders, Hefei, China

**Keywords:** awareness, coma, minimal consciousness state, music, unresponsive wakefulness syndrome (UWS), mismatch negativity (MMN), prediction

## Abstract

**Background:**

Long-term disorders of consciousness (DOC) are a huge burden on both patients and their families. Previously, music intervention has been attempted as a potential therapy in DOC, with results indicating an enhancement of arousal and awareness; yet, to date, there are limited studies on music interventions in DOC with electroencephalogram monitoring. Meanwhile, prediction of awareness recovery is a challenge facing clinicians. The predictive value mismatch negativity (MMN), as a classical cognitive component in event-related potential, is still controversial. In this study, we use auditory event-related potential to probe the effect of music in DOC, and investigate whether music may improve the predictive value of MMN in awareness recovery.

**Methods:**

Fourteen DOC patients were included in the prospective study. Auditory oddball electroencephalogram data were recorded twice with each patient, before and after 5 min of listening to a Chinese symphony that has joyful associations. The outcome was assessed 6 months later.

**Results:**

Significant differences of MMN amplitude were found between healthy controls and pre-music DOC patients (*p* < 0.001), but no significant differences were found between healthy controls and post-music DOC patients. The presence of MMN before music was not correlated with favorable outcome, and 50% of patients with MMN did not recover awareness. When MMN was absent, 50% of patients awoke. After listening to music, among the 11 patients who showed MMN, seven patients recovered awareness. When MMN was absent, no one recovered awareness.

**Conclusions:**

Some DOC patients, even those in a minimal consciousness state and those with unresponsive wakefulness *syndrome (UWS)*, were affected by music. The MMN amplitude was elevated by the music to some extent. A single test of MMN did not have a good prognostic value of our study; however, retesting of MMN after stimulation with familiar music that has joyful associations might be valuable for observation and detection of possible recovery. The musical processing in DOC patients and the effect of musical therapeutic practices need further investigations.

## Background

Craniocerebral trauma, encephalitis, ischemic-hypoxic encephalopathy, and cerebrovascular disease can lead to disorders of consciousness (DOC). The prognosis of these unconscious patients is varied. Some recovered awareness, while others moved into a minimally consciousness state (MCS) (Giacino et al., 2002), even remained in a long-term coma or vegetative state (VS) until death, a term known as unresponsive wakefulness *syndrome* (UWS). Whether and how these individuals are able to regain consciousness is the most important concerns of the patients’ families and clinicians. Evaluation by clinical manifestations alone is usually not sufficient to diagnose consciousness, and the rate of misdiagnosis has been estimated at 37–43% (Hirschberg and Giacino, 2011). In particular, neuroelectrophysiology and neuroimaging technology has shown that clinically diagnosed VS patients retain cortical processing ability. Some studies observed that several MCS or VS patients could answer ‘yes’ and ‘no’ by using activation of different brain regions with the aid of functional magnetic resonance imaging (fMRI) (Owen et al., 2006).

It is noted that the auditory network is one of the reliably observed networks, help to differentiate MCS from VS (Demertzi et al., 2015). Since language disorders were prevalent in people with DOC, language-based assessments and treatments for recovering consciousness and cognition were not sufficient (Schnakers et al., 2014). Acoustic stimuli with characteristics of self-related, personal preferences and emotional valence showed more likely to change with neural activity and behavior (di Stefano et al., 2012; O’Kelly et al., 2013). Thus, the optimal auditory stimuli for assessment and treatment in patients with DOC tend to hold these properties (Magee, 2018). Among the auditory stimuli, music possesses both self-referential properties and emotional valence and can also bypass language (Magee, 2018).

Listening to music has been shown in neuroimaging studies to induce a vast bilateral network of brain activation associated with reward systems, emotions, semantic processing, motor function, and attention in normal subjects (Warren, 2008; Koelsch, 2014) and to influence mood and arousal (Gabriela Husain et al., 2002). Brain activation due to music has also been detected by fMRI in DOC patients (Okumura et al., 2014; Heine et al., 2015), even when no behavioral evidence of language recognition is present (Edlow et al., 2017).

It has been noted that familiar patient preferred music could enhance functional connectivity in people with DOC compared with the white noise condition (Perrin et al., 2015). Brain fMRI data revealed familiar music triggered broad emotion-related areas and reward circuit, showing significant more active than induced by unfamiliar music (Pereira et al., 2011). With familiar objects in an enriched environment, a wider range of behavioral responses was elicited in people with DOC (di Stefano et al., 2012). When some DOC patients listened to familiar preferred music, the EEG power spectrum of frontal midline theta and frontal alpha, as well as respiratory rate and eye blink rate, increased to some extent indicating that there were arousal and selective attention responses (O’Kelly et al., 2013).

Listening to music can also induce long-term plastic changes for the recovery of early processing (Särkämö et al., 2010). After 2 months period of listening to patient preferred music daily, cognitive recovery and mood were enhanced after middle cerebral artery stroke (Särkämö et al., 2008). EEG regional coherence was enhanced on mesocircuit model and thalamocortical synchronization after a 6–18 months music therapy in a DOC case study, which may be an indicator of an increase in consciousness (Lord and Opacka-Juffry, 2016). Thus, listening to familiar patient preferred music is proposed as a beneficial way for people with DOC to improve their perceptual and cognitive abilities and may also be used for prognostic purposes (Magee and O’Kelly, 2015; Perrin et al., 2015). A music therapy assessment tool for awareness in disorders of consciousness (MATADOC) is developed for detecting the awareness and behavior responsiveness to live musical stimuli (Magee et al., 2014). The protocol was a live music based measure delivered by trained board music therapist (Magee et al., 2015). Based on the MATADOC, O’Kelly et al. (2013) reported a VS patient diagnosed by non-music measure improved the assessing score of MCS scale. It seemed that the DOC patients who presented more favorably with music condition would make a better recovery (Magee, 2018). A study with 13 DOC patients found that patients who showed discriminatory event-related potential (ERP) responses to their names after listening to preferred music showed a favorable outcome six months later; meanwhile, no such favorable outcome was found in patients who showed the absence of a discriminative response (Castro et al., 2015).

From studies, music may be effective in assessment and treatment with people with DOC, possibly due to a range of responses on arousal, attention and emotion triggered by music stimuli, which regulates reward pathways and improves neural plasticity (Rollnik and Altenmüller, 2014), thus facilitating awareness of self and environment, irrespective of language, visual, and motor disabilities. To date, however, most of the researches in this area were by neurophysiological and behavior assessment and neuroimaging methods. There is little research on music interventions in DOC patients using EEG. Whether the music can enhance consciousness or cognitive processing even in the absence of behavioral signs in people with DOC need further evidence from EEG.

Event related potentials (ERP) have been used to investigate cognitive processing and cortical learning in DOC patients. It has many advantages, including millisecond (ms) time resolution, high event sensitivity, bedside manipulation, and non-invasive, and had become a valuable clinical investigation tool. Mismatch Negativity (MMN) is an important component of ERP, and a marker of perceptual processing of deviant auditory stimuli representing a form of “primitive intelligence.” In auditory stimuli, MMN appears in the fronto-central area after an infrequent change in a repetitive sequence of sounds (Sams et al., 1985). Auditory oddball paradigms were shown to elicit MMN in patients with DOC (Rodriguez et al., 2014; Wang et al., 2018). MMN can also be used to predict the outcome of comatose patients (Daltrozzo et al., 2007; Faugeras et al., 2011). Since how music effects the MMN in DOC patients is still not clear, here, we used auditory ERP to probe the effect of music on the stimulation of specific areas of the brain in patients with DOC.

It is proposed to use music stimuli familiar to DOC patients for evaluation (Royal College of Physicians, 2013), as well as the patient’s preferred music which have shown more likely to activate arousal and attention relative to patient disliked music (O’Kelly et al., 2013; Pool and Magee, 2016). Meanwhile, compositional features of music stimuli (i.e., tempo, rhythm, melody, harmony, timbre, and loudness) should be considered (Robb et al., 2011). In this study, we chose a Chinese symphony “Spring Festival Prelude” composed by Huanzhi Li in 1956 as the music stimulus. This piece of music was played repeatedly and widely during the Spring Festival in China, which is one the most familiar music to Chinese people and preferred by them. The orchestral music is in C major and F major, allegro, and is associated with scenes of people’s excitement, jubilation, beating drums, singing and dancing in traditional festivals.

We propose that listening to this familiar music with joyful associations can increase attention and arousal in DOC patients, and that the MMN would be enhanced after the music stimuli. The aim of this study was to evaluate MMN performance in these unconscious patients after music exposure and to observe the prognosis.

## Participants and Methods

### Participants

#### DOC Group

From Feb 2016 to Oct 2019, DOC patients admitted to the Neurological Intensive Care Unit of the First Affiliated Hospital of Anhui Medical University were enrolled in this study. The inclusion criteria were: (1) DOC ≥ 14 days of onset and (2) age ≥ 18 years. The exclusion criteria were: (1) history of drug abuse; (2) severe coexisting disease (e.g., acute myocardial infarction, liver and kidney failure, heart failure, or terminal cancer) with a limited likelihood of survival; (3) shock (systolic blood pressure < 80 mmHg); (4) abnormal body temperature; (5) epilepsy; (6) damaged or missing cranial bones; (7) sedatives administered in the previous 24 h; and (8) use of an invasive ventilator or non-invasive ventilator. Patients were recorded without sedation for at least 48 h. Among the 17 recordings three were discarded due to low electroencephalogram (EEG) quality. We included 14 DOC patients (six women), aged from 18–70 years (mean 49.6 years) in this study (Table 1). The etiology of the DOC was ischemic stroke (*n* = 5), anoxic encephalopathy (*n* = 2), acute disseminated encephalomyelitis (ADEM) (*n* = 2), intracranial hemorrhage (*n* = 2), Japanese encephalitis (*n* = 1), hypoglycemic encephalopathy (*n* = 1), and traumatic brain injury (*n* = 1). Twelve patients were diagnosed as an MCS, and two patients were defined as in a VS (Table 1).

**TABLE 1:** Patients’ characteristics and outcomes.

**Patient**	**Gender**	**Age, years**	**Etiology**	**Disease duration, days**	**CRS-R (A-V-M-O-C-Ar)**	**MMN**		**N100**		**6 months follow-up, CRS-R (A-V-M-O-C-Ar)**
		
						**Pre-music**	**Post-music**	**Pre-music**	**Post-music**	
1	F	44	Stroke	15	MCS+ (1, 1, 3, 3, 1, 1)	+	+↑	+	+	Awake (4, 5, 6, 3, 2, 3)
2	M	58	Acute disseminated encephalomyelitis	19	MCS− (0, 2, 2, 0, 0, 1)	-	+↑	+	+	Awake (4, 5, 6, 3, 2, 3)
3	M	43	Anoxicencephalopathy	180	MCS− (2, 2, 1,1, 0, 2)	+	+↑	+	+	MCS− (2, 2, 1,1, 0, 2)
4	F	18	Japanese encephalitis	19	MCS+ (3, 3, 2, 0, 1, 3)	+	+↑	+	+	Awake (4, 5, 6, 0, 2, 3)
5	M	70	Stroke	24	MCS+ (3, 3, 0, 0, 1, 3)	+	+↑	+	+	Awake (4, 5, 5, 0, 2, 3)
6	F	68	Stroke	16	MCS− (2, 3, 2, 0, 1, 2)	+	+	+	+	Awake (3, 5, 6, 1, 2, 3)
7	M	30	Acute disseminated encephalomyelitis	25	MCS− (1, 3, 0, 0, 0, 2)	-	+↑	+	+	Awake (4, 5, 6, 3, 2, 3)
8	F	48	Hypoglycemic encephalopathy	15	UWS (1, 1, 1, 0, 0, 2)	-	+↑	-	+	UWS (1, 1, 1, 0, 0, 2)
9	M	30	Anoxic encephalopathy	31	UWS (1, 1, 1, 0, 0, 2)	-	-	-	-	UWS (1, 1, 1, 0, 0, 2)
10	F	25	Intracranial hemorrhage	150	MCS− (1, 2, 0, 0, 0, 2)	+	-	+	+	MCS− (1, 3, 2, 0, 0, 2)
11	M	60	Traumatic brain injury	180	MCS− (2, 3, 2, 0, 0, 2)	+	+↑	+	+	MCS− (2, 3, 2, 0, 0, 2)
12	F	67	Stroke	15	MCS+ (3, 1, 3, 0, 0, 1)	+	+↑	+	+	Awake (4, 5, 6, 1, 2, 3)
13	M	49	Anoxic encephalopathy	530	MCS− (2, 2, 1,1, 0, 2)	+	-	+	+	MCS− (2, 2, 1, 1, 0, 2)
14	M	63	Stroke	15	MCS− (2, 0,3, 1, 0, 1)	+	+↑	+	+	MCS+ (3, 4, 5, 0, 1, 3)

*CRS-R, Coma Recovery Scale-Revised; CRS-R subscales: A, auditory function; V, visual function; M, motor function; O, oromotor; C, communication; Ar, arousal; ↑, an increase in MMN amplitude; ↓, a decrease in MMN amplitude.*

#### Control Group

Healthy adults (20 in total: eight women, aged 40.0 ± 11.6 years) with no history of drug abuse, mental disorders, or history of brain injury were used as controls.

### Methods

#### Behavior

Neurologic examinations were conducted immediately before ERP. Two trained neurologists evaluated the patients’ state consciousness based on the Coma-Recovery Scale-Revision (CRS-R) (Giacino et al., 2004). A patient who consistently followed instructions correctly, used objects functionally, and used gestures to express “yes” or “no” were defined as aware. MCS patients who showed only minimal levels of behavioral interaction with non-reflex movements, such as visual pursuit or fixation, localization of noxious stimulation, and appropriate emotional response were defined MCS−. A patient who showed reproducible movement to commands, reached for objects, and displayed automatic motor response was defined as MCS+. If the patient showed only clinical signs of unresponsiveness, such as auditory or visual startle, localization to sound or noxious stimulation, flexion withdrawal, abnormal posturing, or no response to the noxious stimulation, that patient was considered to be suffering from UWS, or even worse, was in a coma (Bruno et al., 2011).

We followed each patient for 6 months. The outcomes were categorized as follows: (1) conscious awareness, (2) MCS, (3) UWS or comatose, or (4) deceased. We defined category 1 as favorable outcome and categories 2, 3, and 4 as unfavorable outcome.

### Procedure

**FIGURE 1:**
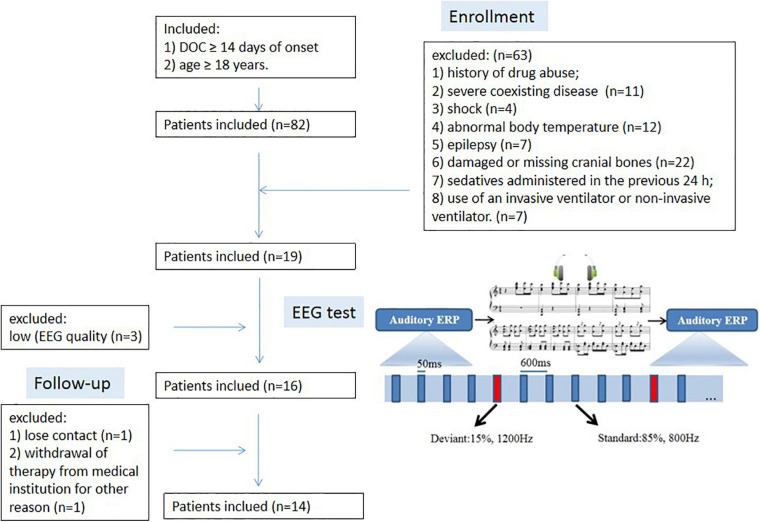
Flow chart of the study.

This study used a modified oddball task. Standard and deviation trials were included in each block (Figure 1), of which 85% were standard trials and 15% were deviation trials (Wijnen et al., 2007). There were three experimental blocks of 200 trials each. The standard stimuli had a tone of 800 Hz and the deviation stimuli had a tone of 1200 Hz. The time difference between the two ears was 0 ms for each stimulus presentation. The procedure used pseudorandom design with at least four standard stimuli between two deviation stimuli. The inter-trial interval was 600 ms. The entire session required about 25 min to complete. The standard trial following the deviation trial was removed to keep the sound group balanced and to ensure habituation of processing standard stimuli. The procedure was presented using E-prime 2.0 software (Psychology *Soft- ware* Tools Inc., Pittsburgh PA, United States).

Electroencephalogram data were recorded twice for each patient. Between the two oddball tasks, patients were allowed to rest for 5 min, and then listened to a Chinese symphony with joyful associations called “Spring Festival Prelude,” which is well known and preferred by Chinese people, for 5 min (Figure 1). The version is included in the album “Golden China” (2008), and played by the China National Symphony Orchestra. The total duration is 5minutes and 2 seconds. The auditory stimuli were delivered by a binaural headphone with 90 dB sound pressure in oddball task and 60–70 dB in music. Through the inquiry of the patients’ close family members, all these patients were familiar with this music, and liked it.

### EEG Data Recording

Electroencephalogram data were recorded from 64 tin electrodes placed on the scalp according to the extended International 10/20 system using a Neuroscan recording system (Neuro Scan, Sterling, VA, United States). EEG signals were recorded using a left mastoid electrode as the online reference. All electrode impedances were maintained below 10 kΩ. EEG activities were amplified with 0.01–100 Hz band-pass filtering and continuously sampled at 500 Hz/channel.

MATLAB scripts using functions from the EEGLAB environment were adopted to process and analyze the EEG data (Delorme and Makeig, 2004). The collected data were re-referenced to the average of the left and right mastoids and were down-sampled to 250 Hz. Then, the data were subjected to a high-pass filter at 1 Hz (FIR filter conducted with pop_eegnewfilt with a default parameters and a cutoff frequency of 0.5 Hz and 26 dB, respectively, to remove baseline drift, thereby ensuring reliable results for independent component analysis) (Delorme et al., 2012). Artifactual channels and non-brain electrodes were rejected by the clean_raw data plugin in EEGLAB, leaving an average of 58.52 (95%, [29, 61]) clean channels per participant. Continuous data were filtered and segmented from 1000 ms before the go and stop signal to 2000 ms after the stimulus. Artifactual epochs were identified and removed based on: (a) abnormal spectral characteristics of high frequency noise (rejspec; 20–40; < −35 or > 35 dB); (b) abnormal trends (rejtrend; slope > 200 μV with R2 > 0.3); (c) abnormal amplitude (threshold −500 μV or + 500 μV); (d) improbable data using joint probability (jointprob, 8 standard deviations (SD) for single channel and 4 SD for all channels); or (e) abnormal distributions (rejkurt; 8 SD for single channel and 4 SD for all channels). Data from electrodes responsible for more than 10% of rejected epochs were discarded. Subsequently, epoch data were decomposed into maximally independent components using an extended infomax algorithm implemented by the runica function with default parameters. Artifactual components electrocardiogram and electromyogram were identified and removed by the EEG_SASICA plugin and visual inspection in EEGLAB (Chaumon et al., 2015). On average, there were 53.56 (95%, [52.8, 54.33]) components left per participant. The mean proportion of rejected epochs was 4.66 (95%, [3.22, 6.09]) in the healthy control group, 5.0 (95%, [2.12, 7.88]) in the pre-DOC group (the DOC group prior to listening to music), and 5.92 (95%, [1.64, 10.21]) in the post-DOC group (the DOC group after listening to music). Rejection rates did not differ significantly among the groups (*F*_2,47_ = 0.26, *p* = 0.77). The cleaned ERP waveforms were time-locked to stimulus onset and epoched to 200 ms pre-stimulus and 1000 ms post-stimulus. The ERPs were averaged separately for standard and deviation trials. N100 was defined as a negative wave between 70–130ms, preceded by a positive deflection P50.

When standard and deviant N100 were detected, the MMN was defined as a negative difference with an average amplitude between 100 and 200 ms. The discriminative MMN wave was accepted when the amplitude was greater than 0.75 uV (Fischer et al., 1999). P3 was defined as the average amplitude between 250-350ms. ERPs data were extracted from the frontal area with F3, FZ, F4, FC3, FCZ, and FC4 electrodes, central area with C3, CZ, and C4 electrodes, and central-parietal area with CP3, CPZ, CP4, P3, PZ and P4 electrodes.

### Statistical Analysis

Quantitative data were presented as mean ± standard errors. Multiple comparisons of MMN amplitude among the groups (control, pre-music DOC, and post-music DOC) were conducted using one-way ANOVA test followed by *post hoc* Dunnett’s T3 test. Multiple repeated ANOVA was conducted with scalp area as within-subject factor, group as between-subject factor. Spearman’s rank test was calculated to test the correlation between CRS-R total scores and MMN amplitudes. χ^2^ test or Fisher’s exact test was applied for categorical variables. Data were analyzed using SPSS software, version 17.0 (SPSS Inc., Chicago, IL, United States).

## Results

### Control Group

In healthy controls, discriminative MMN was present in 20/20 (100%) of subjects. The amplitude elicited by deviation trials was significantly higher (*p* < 0.05) than that elicited by standard trials at 100–200 ms in frontal and central areas. In the frontal area, the MMN measured −5.1 ± 1.8 uV with a latency of 123.8 ± 14.2 ms. In central area, the MMN measured −4.5 ± 1.7 uV with a latency of 120.6 ± 14.9 ms. P3 were elicited with the average amplitude 3.19 ± 2.56 uV at frontal area, 3.256 ± 2.05 uV at central-parietal area.

### DOC Group

In the 14 patients (four MCS+, eight MCS− and two with UWS), discriminative MMNs were evoked in ten subjects (four MCS+ and six MCS−) prior to listening to music, and 11 subjects (four MCS+, six MCS−, and one with UWS) after listening to music listening.

Prior to listening to music, the MMN measured −1.3 ± 2.6 uV with a latency of 122.6 ± 24.6 ms in frontal area, and −1.6 ± 2.1 uV with a latency of 125.4 ± 17.7 ms in central area. Meanwhile, the average P3 measured was −0.95 ± 2.29 uV at frontal area and −0.93 ± 2.24 uV at central-parietal area. After listening to music, the MMN measured was −3.6 ± 3.1 uV with a latency of 117.1 ± 23.7 ms in frontal area and −3.6 ± 3.8 uV with a latency of 127.4 ± 26.9 ms in central area. The average P3 was −0.24 ± 2.22 uV at the frontal area and −0.36 ± 3.5 uV at the central-parietal area.

**FIGURE 2:**
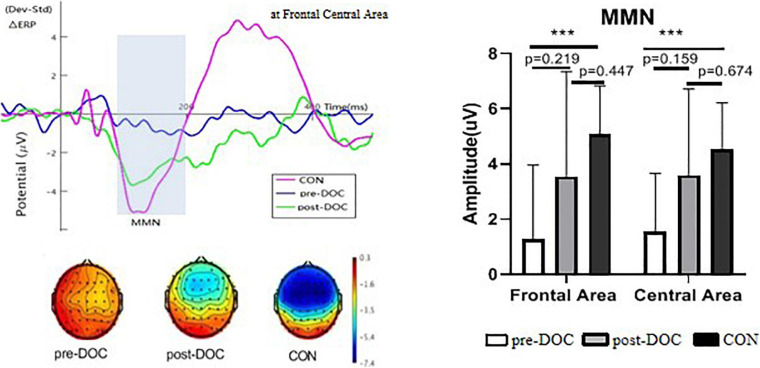
Mismatch negativity response with groups. Data (mean ± SEW) are shown separately for DOC patients, before and after music and controls. ***p < 0.005. Pre-DOC, DOC patents before music; post-DOC, DOC patients after music; CON, healthy controls.

Multiple comparisons of MMN amplitude among the groups (control, pre-music DOC, and post-music DOC) were conducted using one-way ANOVA. Significant differences were found among the groups (*p* < 0.001). A *post hoc* Dunnett’s T3 test showed significant differences between healthy controls and pre-music DOC patients (*p* < 0.001); however, no statistically significant differences was found between healthy controls and post-music DOC patients, or between pre-music DOC patients and post-music DOC patients (Figure 2). Multiple repeated ANOVA was conducted with scalp area (frontal or central) as within-subject factor, group (before or after music) as between-subject factor. There was no within-subjects effect (*F* = 0.19, *p* > 0.05), but the effect between groups was significant (*F* = 7.97, *p* < 0.05), which indicated that music intervention has an influence on MMN in DOC patients. Using the same methods for P3 amplitude analysis, significant difference was found between healthy control and pre-music DOC patients (*p* < 0.05), as well as between healthy control and post-music DOC patients (*p* < 0.05). The P3 amplitude had no significant difference between pre-music DOC patients and post-music DOC patients (*p* > 0.05). No within-subject effect or between-group effect was found by multiple repeated ANOVA (*p* > 0.05).

There was no association between total CRS-R scores and the amplitude of MMN, both prior to and after listening to music (*p* > 0.05). The correlation between the changes of MMN amplitudes (before and after music) with CRS-R total scores did not reach the significance (*p* = 0.15). The presence of MMN before music was not linked to favorable patient outcome at the 6 months follow-up (Fisher exact test, *p* > 0.05), with sensitivity of 71%, specificity of 28.6%, a positive predictive value of 50%, and negative predictive value of 50%. Before music, when MMN was present, 5/10 awoke. When MMN was absent, 2/4 awoke. After listening to music, among the 11 patients who showed MMN after listening to music, seven recovered awareness. When the MMN was absent, 3/3 (100%) did not recover awareness. The total predictive value of MMN after the music did not reach to the statistical significance (Fisher’s exact test, *p* > 0.05), but with a sensitivity of 100%, and specificity of 42.9%. Positive predictive value was 63.6% and a negative predictive value was 100%. We further used chi-square to evaluate the value of the presence of N100, as well as the combination of MMN and N100 for prognosis. However, both of them did not reach to significant value in the prognosis (Fisher’s exact test, *p* > 0.05). Meanwhile, no statistical relationship was established between the presence of increased MMN and a favorable outcome that recovered conscious awareness (Fisher’s exact test, *p* > 0.05). In this study, nine patients (9/14) had increased MMN after listening to music, six of which (6/9) recovered conscious awareness; however, five patients (5/14) showed no increases in MMN, four of which had an unfavorable outcome.

## Discussion

Mismatch negativity is the earliest cognitive component in an ERP trace. MMN presents a negative deflection on frontal and central areas, with a latency of between 100 and 250 ms after deviance onset (Sams et al., 1985). MMN links to fresh-afferent neuronal activity, reflecting the brain’s ability to determine automatic comparisons (Garrido et al., 2009). The generation of MMN might involve an index of memory and an involuntary attention switching process (Garrido et al., 2009). Furthermore, MMN can be elicited below the level of attention, even in sleep and coma.

It has been shown that the frontal cortex and parietal cortex are involved in conscious perception (Del Cul et al., 2007; Koch et al., 2016). DOC patients who elicit an MNN are considered to be in a semi-conscious state, and deficits in MMN are associated with DOC (Faugeras et al., 2011). In our study, MMN was found to be elicited in the frontal and central areas. Meanwhile, the average amplitude of MMN in DOC patients was lower than healthy controls, although no association was found between total CRS-R scores and the amplitude of MMN.

Several studies have indicated that music has been shown to elicit neural responses, altering cortical activity and connectivity both in healthy subjects and clinically “unresponsive” patients (Wilkins et al., 2014). Music therapy appears to be a promising clinical tool for rehabilitation of DOC patients, as well as dementia patients (Sihvonen et al., 2017). Music’s features with patients, such as autobiographical re-experiencing, familiarity, and preference, would have beneficial effects (O’Kelly et al., 2013; Castro et al., 2015). In this study, we chose a Chinese symphony with known joyful associations called “Spring Festival Prelude” which was familiar to the subjects and liked by them. The musical excerpt was selected based on the musical stimuli using in prior studies with properties of familiar, preferred, and emotional valence. And it also meets the characteristics of frequent changes in tempo, dynamic, musically coherent and representative of the whole musical piece (Castro et al., 2015), such as Aaron Copland’s “Rodeo – Four Dance Episodes” and the first 16 measures of “Les Toreador” from “Carmen” Suite No.1 by Bizet that used in previous studies (Danielsen et al., 2014; Okumura et al., 2014).

The MMN amplitude was also elevated by music to some extent. Nine of the 14 (64%) patients had an increased MMN amplitude after listening to music. There was a statistically significant difference of MMN amplitude between DOC patients before listening to music and healthy controls. But after listening to music, no statistical difference existed between DOC patients and healthy controls. Analysis by multiple repeated ANOVA also indicated that music intervention has an influence on MMN in DOC patients. We therefore considered that many DOC patients, even those who were MCS− and those suffering from UWS, were affected by listening to the music, which in turn improved MMN—a factor considered to be a marker of consciousness involved in acoustic discrimination and sensory memory. As for the improved, MMN is a basis of the emerging conscious perception and awareness, it is rational to use music-intervention for promoting consciousness recovery.

P300 is an endogenous component of ERP, which is related to the cognitive process, attention, memory, intelligence and mental state of the brain. But the sensitivity of the P300 elicited in a short two-tone oddball paradigm was too low in VS and MCS patients for application in a clinical setting (Real et al., 2016). Similar to the previous study, P300 did not elicit well in our patients with DOC when using the two pure tones oddball paradigm, which limited the use of P300 for prognosis. While complex sensory tones, such as subject’s own name, familiar sound could induce larger P300 and had predictive value in DOC patients (Daltrozzo et al., 2007) and could be used in a further study.

Mismatch negativity had been used in the prognosis of awareness. Despite having good specificity, MMN was found to have a poor sensitivity and detection rate. A meta-analysis of MMN for prediction of awareness showed a sensitivity of 38%, specificity of 91%, positive predictive value of 88%, negative predictive value of 46% (Daltrozzo et al., 2007). Fisher et al. studied 128 comatose patients, and on the 8th day after average coma, only 33 patients elicited MMN. Thirty of the 95 patients who recovered awareness showed an MMN (31.6% sensitivity) (Fischer et al., 1999). A study of 346 comatose patients found that when MMN was present (25.4%), 88.6% patients awoke, and when it was absent, 62.4% patients did not (Fischer et al., 2004).

In our study, 10 patients who elicited an MMN, half regained awareness prior to the 6 months follow-up, while two of the four the patients that did not elicit an MMN recovered their awareness. In regard to prognostic value, the presence of MMN before listening to music was not correlated with favorable patient outcome, with sensitivity of 71%, specificity of 28.6%, a positive predictive value of 50%, and negative predictive value of 50%. The specificity of the baseline MMN is relatively low compared with the high specificity validated in previous studies in literature. We noted that the duration of disease and the etiology to the coma may confound the results. Within the five patients who did not recover awareness but had MMN, four of them had the EEG test exceeding 6 months after the onset of the coma. Both of the two patients who had no MMN but then regained consciousness were not old and diagnosed as ADEM with predominant white matter damages, which is generally better in prognosis than other causes. Meanwhile, the time of the EEG test in these two ADEM patients was less than one month after onset.

After listening to music, among the 11 patients who elicited an MMN, seven patients recovered awareness prior to the 6 months follow-up. However, three patients who did not elicit MMN after listening to music remained unconscious at 6 months follow-up. We also noted that there were three patients who recovered awareness that did not initially elicit MMN, but did so after listening to music. There were still two patients who showed MMN first but did not induce MMN after the music, and they did not regain consciousness. As a result, the prognostic value of MMN after music seemed to increase than before music, with a sensitivity of 100% vs. 71%, specificity of 42.9% vs. 28.6%, positive predictive value of 63.6% vs. 50%, and a negative predictive value 100% vs. 50%. Thus, retesting of MMN after stimulation with familiar music that has joyful associations may be a good attempt for the observation and detection of possible recovery. However, increased MMN after the music did not prove to be linked with favorable outcome (*p* = 0.19).

There are some limitations of our study. The sample size used in the present study was small, which limited generalization of the conclusions. Many factors may be the confounds of the study, such as the cause of the initial disease, the injured region, a patient’s age, disease duration, the delays between the EEG test and the onset of the coma, which should be taken into account in future studies. The MMN we used is far from being a proof of the subclinical consciousness, and we did not elicit P300 well. The auditory oddball paradigm we used need to be modified, and other paradigms such as novelty P3 elicited by the subject’s own name, and motor imagery tasks can be used further.

## Conclusion

In conclusion, we considered that many DOC patients, even those who were found to be MCS− and who suffered from UWS, could be positively affected by music. In particular, the MMN amplitude was elevated by the music to some extent, so it is reasonable to use music-intervention for promoting the recovery of consciousness. A single test of MMN did not have a good prognostic value in our study; however, retesting of MMN after stimulation with familiar music that has joyful associations may be valuable for observation and detection of possible recovery.

The musical processing in DOC patients and the effect of musical therapeutic practices need further investigation. Larger samples are needed to prove the predictive value of MMN after music with specific etiology of DOC in a long-term clinical follow-up.

## Data Availability Statement

The raw data supporting the conclusions of this article will be made available by the authors, without undue reservation.

## Ethics Statement

The studies involving human participants were reviewed and approved by The First Affiliated Hospital of Anhui Medical University, Hefei. The patients/participants provided their written informed consent to participate in this study.

## Author Contributions

YH and FY designed the experiments. YH, XY, and CW carried out the experiments. FY and YH analyzed the experimental results. YH and FY wrote the manuscript. KW contributed to the manuscript writing, review, and supervision. All authors contributed to the article and approved the submitted version.

## Conflict of Interest

The authors declare that the research was conducted in the absence of any commercial or financial relationships that could be construed as a potential conflict of interest.
